# L-Cystathionine Protects against Homocysteine-Induced Mitochondria-Dependent Apoptosis of Vascular Endothelial Cells

**DOI:** 10.1155/2019/1253289

**Published:** 2019-11-25

**Authors:** Xiuli Wang, Yi Wang, Lulu Zhang, Da Zhang, Lu Bai, Wei Kong, Yaqian Huang, Chaoshu Tang, Junbao Du, Hongfang Jin

**Affiliations:** ^1^Department of Pediatrics, Peking University First Hospital, Beijing 100034, China; ^2^Research Unit of Clinical Diagnosis and Treatment of Pediatric Syncope and Cardiovascular Diseases, Chinese Academy of Medical Sciences, Beijing, China; ^3^Department of Physiology and Pathophysiology, Peking University Health Science Center, Beijing 100191, China; ^4^Key Lab. of Ministry of Education of China, Beijing 100191, China

## Abstract

The study was aimed at investigating the effects of L-cystathionine on vascular endothelial cell apoptosis and its mechanisms. Cultured human umbilical vein endothelial cells (HUVECs) were used in the study. Apoptosis of vascular endothelial cells was induced by homocysteine. Apoptosis, mitochondrial superoxide anion, mitochondrial membrane potential, mitochondrial permeability transition pore (MPTP) opening, and caspase-9 and caspase-3 activities were examined. Expression of Bax, Bcl-2, and cleaved caspase-3 was tested and BTSA1, a Bax agonist, and HUVEC Bax overexpression was used in the study. Results showed that homocysteine obviously induced the apoptosis of HUVECs, and this effect was significantly attenuated by the pretreatment with L-cystathionine. Furthermore, L-cystathionine decreased the production of mitochondrial superoxide anion and the expression of Bax and restrained its translocation to mitochondria, increased mitochondrial membrane potential, inhibited mitochondrial permeability transition pore (MPTP) opening, suppressed the leakage of cytochrome c from mitochondria into the cytoplasm, and downregulated activities of caspase-9 and caspase-3. However, BTSA1, a Bax agonist, or Bax overexpression successfully abolished the inhibitory effect of L-cystathionine on Hcy-induced MPTP opening, caspase-9 and caspase-3 activation, and HUVEC apoptosis. Taken together, our results indicated that L-cystathionine could protect against homocysteine-induced mitochondria-dependent apoptosis of HUVECs.

## 1. Introduction

Homocysteine (Hcy) is an important sulfur-containing amino acid. The concentration of Hcy over 15 *μ*mol/L in plasma is defined as hyperhomocysteinemia [1]. Hcy is an independent risk factor for cardiovascular disease and can cause the damage to vascular endothelial cells (VECs), thus participating in the pathogenesis of a variety of diseases including atherosclerosis, hypertension, and coronary artery disease [2–5]. Previous studies showed that high homocysteine accentuated the production of reactive oxygen species, thereby activating the mitochondrial apoptotic pathway resulting in an apoptosis of VECs, which is regarded as an important cause of vascular dysfunction [6].

In order to treat hyperhomocysteinemia and reduce the incidence of cardiovascular disease, many studies have been conducted in this field. In the past, it was considered that the deficiency of vitamin B complex was an important factor in the formation of hyperhomocysteinemia. Therefore, the method of supplementing folic acid, vitamin B6, and vitamin B12 was used to reduce the concentration of homocysteine in the body, but clinical studies have shown that although vitamin B complex reduces homocysteine concentration in plasma, it cannot significantly inhibit Hcy-induced endothelial dysfunction [7–9]. Thus, it is necessary to develop new modalities of medication to prevent endothelial dysfunction and cardiovascular events caused by Hcy.

L-cystathionine is a nonprotein thioether containing amino acids and is mainly produced in the metabolic transformation process of methionine to cysteine in the body [10]. Previous studies on L-cystathionine have focused on it as a key amino acid associated with the metabolic state of sulfur-containing amino acids [11]. In recent years, studies have shown that it plays an important role in cardiovascular protection other than merely a metabolite in the methionine metabolic pathway. For example, L-cystathionine can antagonize vascular injury through the inhibition of ox-LDL-induced inflammatory response in macrophages [12]. Nevertheless, how L-cystathionine regulates Hcy-induced vascular endothelial cell injury remains unknown.

Therefore, this present study was undertaken to investigate the protective effect of L-cystathionine on Hcy-induced VEC apoptosis and reveal the significance and mechanism by which L-cystathionine maintains VEC homeostasis.

## 2. Materials and Methods

### 2.1. Cell Culture and Processing

Human umbilical VECs (HUVECs) were purchased from the China Infrastructure of Cell Line Resources Centre. The cells were grown in DMEM containing 10% FBS, 1% penicillin, 1% streptomycin, and 1% glutathione and maintained in an incubator at 37°C with 5% CO_2_. When the cell confluence reached 80%, the experiments were started. Before each experiment, the cells were placed in synchronization buffer for 12 h. Cells were divided into the control group, Hcy group, Hcy+0.1 mmol/L L-Cth group, Hcy+0.3 mmol/L L-Cth group, and Hcy+1.0 mmol/L L-Cth group. According to the manufacturer's instructions, L-cystathionine was dissolved in 0.5 mol/L hydrochloric acid to a stock concentration of 5 mg/mL (22.5 mmol/L) in this study. Cells in the Hcy+0.1 mmol/L L-Cth group, Hcy+0.3 mmol/L L-Cth group and Hcy+1.0 mmol/L L-Cth group were pretreated with L-cystathionine for 30 min and then stimulated with 500 *μ*mol/L Hcy for 24 h. Cells in the Hcy group were treated with 500 *μ*mol/L Hcy for 24 h [13]. For the purpose of Bax manipulation, cells were preadministered with 0.625 *μ*mol/L BTSA1 for 2.5 h [14], followed by 0.3 mmol/L L-cystathionine and 500 *μ*mol/L homocysteine treatment. To further understand the role of Bax expression in the protection of cell apoptosis by L-cystathionine, the cells were transfected with 1 *μ*g of vehicle plasmid or bax-overexpressed plasmid. Freshly completed culture medium was replaced after 6 h of transfection, and synchronization was performed after another 24 h, and then L-cystathionine and homocysteine were sequentially added.

### 2.2. *In Situ* and Quantitative Detection of Apoptosis in HUVECs by Using TdT-Mediated dUTP Nick End Labeling (TUNEL) Assay and ELISA

*In situ* cell apoptosis was determined with an *in situ* cell death detection kit and fluorescein (R&D Systems, USA) in accordance with the instructions of the manufacturer. Briefly, the cells on slides were fixed in 4% paraformaldehyde for 15 min after washing three times with PBS. Then, the cells were incubated with permeabilization solution at 37°C for 30 min. After washing with PBS, the cells were incubated with TUNEL reaction mixture for 60 min at 37°C in the dark. The antifade solution was used to mount the slides after washing three times with PBS, and the slides were analyzed under a confocal laser scanning microscope (Olympus, Japan).

Moreover, the quantitative detection of DNA fragments in HUVECs was measured with a Cell Death Detection ELISA^PLUS^ Kit (Roche, Mannheim, Germany). According to the manufacturer's instructions, an appropriate volume of cell lysis buffer was added to lyse the cells. The cell lysate was added into a streptavidin-coated microplate. A mixture of anti-histone-biotin and anti-DNA-POD was added and incubated. The microplate was vortexed at room temperature for 2 h. The unbound components were removed after washing three times with incubation buffer, and an appropriate volume of substrate solution was added to each well. The microplate was vortexed at room temperature for 20 min, and the reaction was stopped by the addition of a stop solution. A microplate reader was used to obtain an absorbance value of each well, and the apoptosis level was calculated [15].

### 2.3. Detection of Mitochondrial Superoxide Anion by the MitoSOX Reagent in HUVECs

A MitoSOX Red Mitochondrial Superoxide Indicator (Life Technologies, USA) was used to measure mitochondrial ROS production. The indicator was applied to incubate the treated cells at 37°C for 10 min, protected from light. After washing with PBS, the cells were fixed in prewarmed 4% paraformaldehyde at room temperature for 15 min after washing with warm PBS for three times. The slides were mounted with an antifluorescence quencher (Beijing Zhongshan Golden Bridge Biotechnology Company, Beijing, China) after washing with PBS. Then, the cells on slides were detected with a laser scanning confocal microscope (Olympus, Japan).

### 2.4. Assessment of Cell Viability in HUVECs

The CCK8 assay was used to evaluate the cell viability in HUVECs (Beyotime, Shanghai, China). The cells were seeded in a 96-well plate. After the treatment with Hcy alone or Hcy plus L-cystathionine, CCK8 solution was added and incubated with cells for 2 h at 37°C. A microplate reader (Thermo, Finland) was used to detect the absorbance at a wavelength of 450 nm.

### 2.5. Measurement of Lactate Dehydrogenase (LDH) Activity in the Culture Media

LDH activity in the culture media of the HUVECs was measured with an LDH cytotoxicity assay kit (Beyotime, Shanghai, China). The cells were seeded in a 96-well plate. After the treatment with Hcy alone or Hcy plus L-cystathionine, LDH activity analysis was carried out according to the manufacturer's instructions. The absorbance of each well was read at 490 nm with a microplate reader.

### 2.6. Measurement of Mitochondrial Membrane Potential in HUVECs

Mitochondrial membrane potential changes in HUVECs were detected with a JC-1 mitochondrial membrane potential detection kit (Beyotime, Shanghai, China). When the mitochondrial membrane potential is at a high level, JC-1 accumulates within the mitochondria and becomes fluorescent red. When the mitochondrial membrane potential is at a low level, JC-1 cannot accumulate within the mitochondria and remains in the cytoplasm in a green fluorescent monomeric form. A 1 : 1 mixture of JC-1 staining working solution and cell culture medium was added to cover the cell culture slides and incubated at 37°C in the dark for 20 min. The slides were washed twice with JC-1 assay buffer and fixed with 4% paraformaldehyde at room temperature for 20 min. Then, the slides were washed three times with PBS and mounted with antifade mounting media. The cells were analyzed with a confocal laser scanning microscope (Olympus, Japan).

### 2.7. Detection of Mitochondrial Permeability Transition Pore (MPTP) Opening in HUVECs

The MPTP opening in HUVECs was determined with a Cell MPTP Assay Kit (Genmed Scientific Inc., Arlington, TX, USA). Calcein AM, as a fluorescent probe, enters the mitochondria to form a green fluorescent compound. Once the mitochondrial permeability transition pore is opened, calcein AM is released, and accordingly, the fluorescence is quenched. First, culture medium was discarded and slides were rinsed gently with cleaning solution. Slides were subsequently incubated with the staining working solution at 37°C in the dark for 30 min. 4% paraformaldehyde was used to fix the cells for 15 min after rinsing for three times with cleaning solution. Finally, the slides were washed 3 times with PBS and mounted with antifade mounting media. A laser scanning confocal microscope was used for the analysis.

### 2.8. Immunofluorescence Microscopy

To determine the localization of cytochrome c or Bax, the cells were incubated in prewarmed medium containing 200 nmol/L of MitoTracker (Life Technologies, USA) at 37°C for 30 min after washing twice with PBS, and then, 4% paraformaldehyde was used to fix the cells at room temperature for 15 min. After washing three times with PBS, the cells were permeabilized with 0.1% Triton X-100 and then stained with anti-cytochrome c or anti-Bax at room temperature for 1 h and at 4°C overnight. After the incubation, an Alexa 594-conjugated secondary antibody (Life Technologies, USA) was added and incubated at 37°C for 90 min. Then, the antifade mounting media were used to mount the slides, and the cells were imaged with a confocal laser scanning microscope (Olympus, Japan) [16].

### 2.9. Measurement of Caspase-9 Activities in Human Macrophages by a Fluorescence and Colorimetric Assay

Change of *in situ* caspase-9 activity in HUVECs was detected with a living cell caspase-9 Fluo-staining kit (Genmed Scientific Inc., Arlington, TX, USA). The cell culture slides were rinsed with wash buffer and incubated with the staining working solution at 37° C in the dark for 20 min. After washing three times with wash buffer, fixation buffer was used to fix the cells at room temperature for 30 min. Then, antifade mounting media were used to mount the slides and the cells were analyzed with a confocal laser scanning microscope (Olympus, Japan).

Caspase-9 activity in HUVECs was detected with a cell caspase-9 activity colorimetric kit (Applygen, Beijing, China). Briefly, after washing twice with the wash buffer, the cells were incubated with Applygen lysis buffer at 4°C for 30 min and then harvested into a centrifuge tube by scraping. The tubes were centrifuged (12,000 × *g*) at 4°C for 5 min. The supernatant was collected in a fresh tube on ice. The Bradford method was used for protein quantification. Then, 50 *μ*g of sample and a reaction reagent were added to each well of a 96-well plate in order and incubated at 37°C in the dark for 2 h. A microplate reader at 405 nm was used to obtain an absorbance value of each well, and the caspase-9 activity level was calculated.

### 2.10. Measurement of Caspase-3 Activities in Human Macrophages by a Colorimetric Assay

Caspase-3 activity in HUVECs was quantified using a cell caspase-3 activity colorimetric kit (Applygen, Beijing, China). The cells were scraped on ice with Applygen lysis solution after washing twice with PBS. The supernatant was transferred into tubes after centrifugation (12,000 × *g*) at 4°C for 5 min. The Bradford method was used to measure protein concentration. The protein and reagent were added in a 96-well plate in order and incubated for 2 h at 37°C in the dark. Finally, the 96-well plate was read in a microplate reader.

### 2.11. Preparation of Mitochondrial Protein

A mitochondria isolation kit (Beyotime, Shanghai, China) was used to extract mitochondrial and cytosolic protein. The HUVECs were mixed thoroughly with Mito solution after centrifugation (600 × *g*) at 4°C for 5 min. After incubating on ice for 30 min with Mito solution, grinding pestles were used to grind the cells. Then, the cells were transferred to centrifuge tubes. The supernatant was moved into precooled tubes after centrifugation (1000 × *g*) at 4°C for 10 min. The supernatant was moved into new precooled tubes after centrifugation (3500 × *g*) at 4°C for 10 min. The supernatant containing the cytoplasm was then collected after centrifugation (12,000 × *g*) at 4°C for 10 min, while the precipitate containing mitochondria was resuspended with Mito lysate. Mitochondrial and cytosolic protein concentration was measured by Bradford methods.

### 2.12. Western Blotting Analysis

Western blotting was used to determine the expression of Bcl-2, Bax, cytc, flag, and cleaved caspase-3 and caspase-3 in HUVECs. The cells were lysed with an appropriate volume of protein lysis buffer at 4°C for 20 min after washing twice with precooled PBS. The lysate was transferred to an Eppendorf tube after fully scraping the cells. The tubes were centrifuged (12,000 × *g*) for 10 min, and the supernatant was collected. A small volume of supernatant was used for protein quantification (Bradford method). The remaining supernatant was boiled in equal volume of 2× sample buffer at 100°C for 10 min. Protein samples (30 *μ*g) were separated in 10% SDS-PAGE and then electrically transferred onto a nitrocellulose membrane with a constant current of 200 mA for 2 h. After the transfer, the nitrocellulose membrane was blocked with 5% skim milk for 1 h. Then, the membrane was incubated with the following primary antibodies: *β*-actin (Santa Cruz, USA), Bcl-2 (Cell Signaling Technology, USA), Bax (Cell Signaling Technology, USA), cytc (Santa Cruz, USA), caspase-3 (Beyotime, Shanghai, China), cleaved caspase-3 (Beyotime, Shanghai, China), cytochrome c oxidase IV (Cell Signaling Technology, USA), and flag (ZSBiO, China) at room temperature for 2 h and at 4°C overnight. Cytochrome c oxidase IV (COX IV) and *β*-tubulin were used as the markers of mitochondrial and cytosolic protein, respectively. After the incubation, a horseradish peroxidase- (HRP-) conjugated secondary antibody was added and incubated at room temperature for 1 h. The protein band images were developed with FluorChem M (Protein Simple, USA) and quantitatively analyzed with the AlphaImager graphical analysis software (Alpha Innotech Corporation, USA).

### 2.13. Statistical Analysis

The quantitative data are expressed as the mean ± SEM. SPSS 23.0 was used for data analysis. The mean among the three groups was compared by ANOVA. If the homogeneity of the variance test showed equal variance, the Bonferroni test was used to compare the differences between two groups. Otherwise, Dunnett's T3 test was used to compare the differences between two groups. *P* < 0.05 was considered statistically significant.

## 3. Results

### 3.1. L-Cystathionine Inhibited Hcy-Induced Cytotoxicity in HUVECs

**Figure 1:**
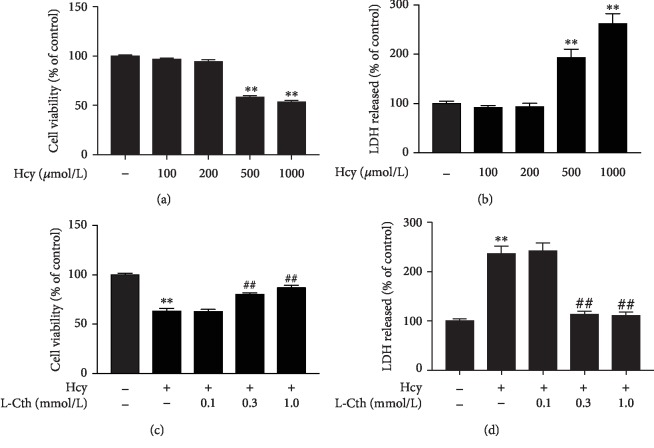
Effects of L-Cth on Hcy-induced cytotoxicity in HUVECs. (a) Cell viability in HUVECs treated with different concentrations of Hcy was measured by the CCK8 assay. (b) The LDH release from HUVECs treated with different concentrations of Hcy was analyzed. (c) Cell viability in HUVECs treated with 500 *μ*mol Hcy alone or Hcy plus different concentrations of L-Cth was measured by the CCK8 assay. (d) The LDH release from HUVECs treated with 500 *μ*mol Hcy alone or Hcy plus different concentrations of L-Cth was analyzed. Data are presented as mean ± SEM. ^∗∗^*P* < 0.01 versus control group; ^##^*P* < 0.01 versus Hcy group. L-Cth: L-cystathionine; LDH: lactate dehydrogenase.

The CCK8 assay and LDH leakage assay were used to evaluate the cytotoxicity of Hcy on HUVECs. As shown in Figures 1(a) and 1(b), the treatment of HUVECs with 500 and 1000 *μ*mol/L Hcy significantly suppressed the cell viability and increased the LDH activity in the culture medium. In contrast, pretreatment of HUVECs with 0.3 and 1.0 mmol/L L-cystathionine significantly increased cell viability and blocked the LDH release in the HUVECs treated with 500 *μ*mol/L Hcy (Figures 1(c) and 1(d)). These results suggested that L-cystathionine could inhibit Hcy-induced cytotoxicity in HUVECs.

### 3.2. L-Cystathionine Suppressed Cell Apoptosis Induced by Hcy in HUVECs

**Figure 2:**
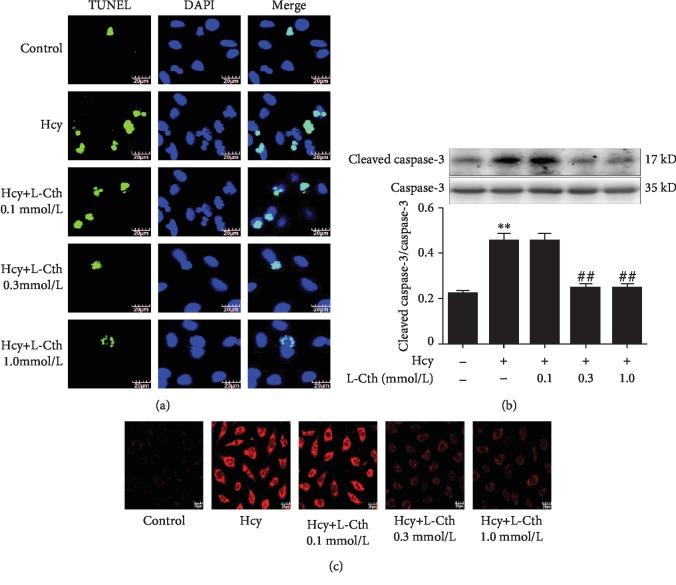
Effects of L-Cth on apoptosis, caspase-3 activities, and mitochondrial superoxide generation in Hcy-treated HUVECs. (a) HUVEC apoptosis detected by TdT-mediated dUTP nick end labeling (TUNEL) methods (magnification, ×600; scale bar: 20 *μ*m). (b) Cleavage of caspase-3 analyzed by western blotting. (c) Mitochondrial superoxide generation in HUVECs detected with MitoSOX. Data are presented as mean ± SEM. ^∗∗^*P* < 0.01 versus control group; ^##^*P* < 0.01 versus Hcy group. L-Cth: L-cystathionine.

To study whether L-cystathionine could inhibit cell apoptosis induced by 500 *μ*mol/L Hcy in HUVECs, we used the TUNEL assay to detect cell apoptosis. Results showed that the green fluorescence intensity was significantly higher in the Hcy group than in the control group, indicating that Hcy could significantly increase the apoptosis of the cells. After the treatment with 0.1 mmol/L L-cystathionine, no significant change in apoptosis was observed. However, the treatment with 0.3 and 1.0 mmol/L L-cystathionine significantly inhibited Hcy-induced apoptosis, respectively (Figure 2(a)).

Western blotting was used to analyze the cleavage of caspase-3 protein. Data showed that 500 *μ*mol/L Hcy significantly promoted caspase-3 cleavage. Pretreatment with 0.1 mmol/L L-cystathionine did not alter the expression of cleaved caspase-3 protein, while 0.3 and 1.0 mmol/L L-cystathionine significantly inhibited the cleavage of caspase-3, respectively. These results demonstrated that L-cystathionine antagonized apoptosis induced by Hcy in HUVECs (Figure 2(b)).

### 3.3. L-Cystathionine Inhibits Mitochondrial Superoxide Anion Generation Induced by Hcy in HUVECs

Results showed that the red fluorescence intensity was significantly higher in the Hcy group than in the control group, indicating that mitochondrial superoxide generation in the Hcy group was significantly increased compared with that in the control group. Pretreatment with 0.1 mmol/L L-cystathionine did not change mitochondrial superoxide generation. However, when pretreated with 0.3 and 1.0 mmol/L L-cystathionine, the generation of mitochondrial superoxide dramatically declined (Figure 2(c)).

### 3.4. L-Cystathionine Inhibits the Expression of Bax Induced by Hcy in HUVECs

**Figure 3:**
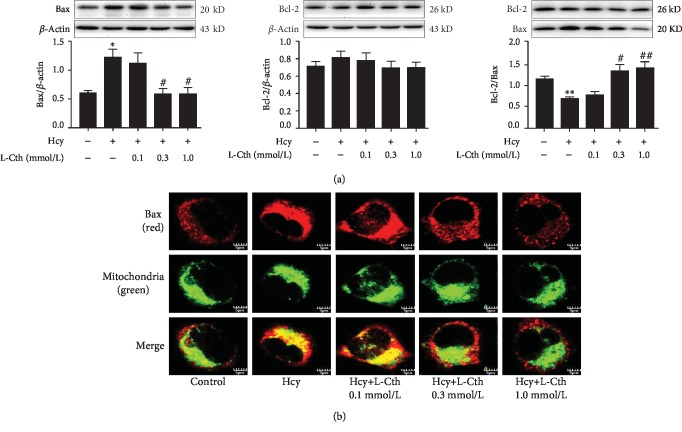
Effect of L-Cth on Bax and Bcl-2 expression as well as Bax distribution in Hcy-treated HUVECs. (a) Bax and Bcl-2 expression analyzed by western blotting. Data are presented as mean ± SEM. ^∗^*P* < 0.05 versus control group; ^∗∗^*P* < 0.01 versus control group; ^#^*P* < 0.05 versus Hcy group; ^##^*P* < 0.01 versus Hcy group. (b) The localization of Bax in HUVECs detected by immunofluorescence microscopy, with red fluorescence indicating Bax and green fluorescence indicating mitochondria (magnification, ×600; scale bar: 5 *μ*m). L-Cth: L-cystathionine.

Western blotting results showed that 500 *μ*mol/L Hcy significantly increased Bax protein expression but did not impact on Bcl-2 protein expression, resulting in a decrease in the ratio of Bcl-2/Bax, which activated the mitochondrial pathway to cause apoptosis. However, pretreatment with 0.3 and 1.0 mmol/L L-cystathionine significantly inhibited the increased Bax protein expression, respectively, while the Bcl-2 protein expression was unaffected, which increased the ratio of Bcl-2/Bax and antagonized apoptosis. Pretreatment with 0.1 mmol/L L-cystathionine had no impact on Bax and Bcl-2 protein expressions (Figure 3(a)). Moreover, immunofluorescence microscopy was used to determine the localization of Bax. Results showed that 0.3 and 1.0 mmol/L L-cystathionine not only reduced the expression of Bax but also inhibited its translocation to mitochondria, respectively (Figure 3(b)).

### 3.5. L-Cystathionine Reversed the Decline of Mitochondrial Membrane Potential Induced by Hcy in HUVECs

**Figure 4:**
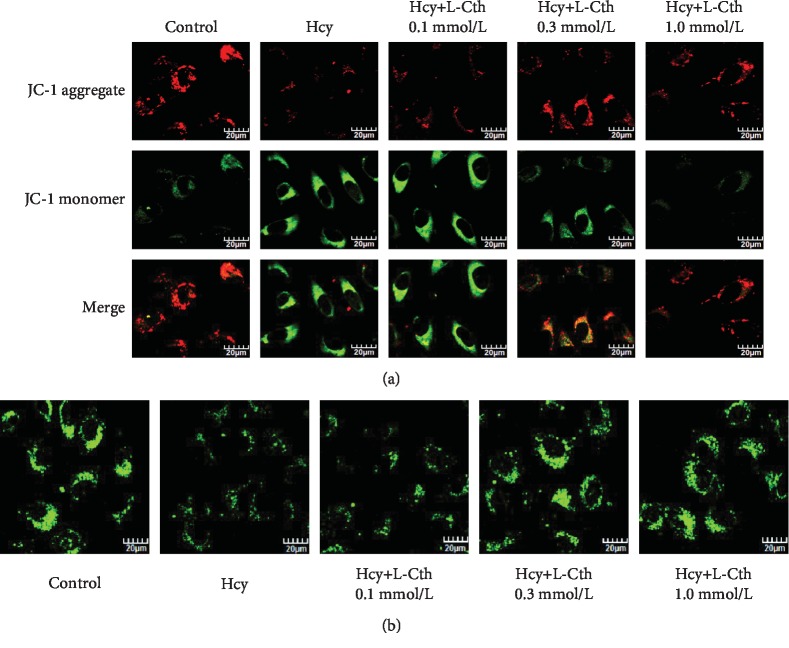
Effect of L-Cth on mitochondrial membrane potential and mitochondrial permeability transition pore (MPTP) opening in Hcy-treated HUVECs. (a) Change of mitochondrial membrane potential detected by a JC-1 fluorescent probe, with red fluorescence indicating high mitochondrial membrane potential and green indicating low mitochondrial membrane potential (magnification, ×600; scale bar: 20 *μ*m). (b) Changes of MPTP opening in HUVECs detected with calcein AM. The green fluorescence quenching represented MPTP opening (magnification, ×600; scale bar: 20 *μ*m). L-Cth: L-cystathionine.

The green fluorescence intensity was significantly higher in the Hcy group than in the control group, indicating that 500 *μ*mol/L Hcy could reduce the mitochondrial membrane potential. After the addition of 0.1 mmol/L L-cystathionine, there was no significant change in the mitochondrial membrane potential. However, the red fluorescence intensity was significantly enhanced after the addition of 0.3 and 1.0 mmol/L L-cystathionine, respectively. These results indicated that 0.3 and 1.0 mmol/L L-cystathionine could reverse the decline of mitochondrial membrane potential induced by Hcy (Figure 4(a)).

### 3.6. L-Cystathionine Antagonized Mitochondrial Permeability Transition Pore (MPTP) Opening Induced by Hcy in HUVECs

Compared with the control group, fluorescence intensity was significantly weakened in the Hcy group, suggesting an increased MPTP opening. However, a significantly enhanced fluorescence intensity was observed when pretreated with 0.3 and 1.0 mmol/L L-cystathionine, respectively, implying the inhibition of MPTP opening. When HUVECs were pretreated with 0.1 mmol/L L-cystathionine, however, fluorescence intensity did not alter (Figure 4(b)).

### 3.7. L-Cystathionine Inhibited the Release of Cytc from the Mitochondrion into the Cytoplasm Induced by Hcy in HUVECs

**Figure 5:**
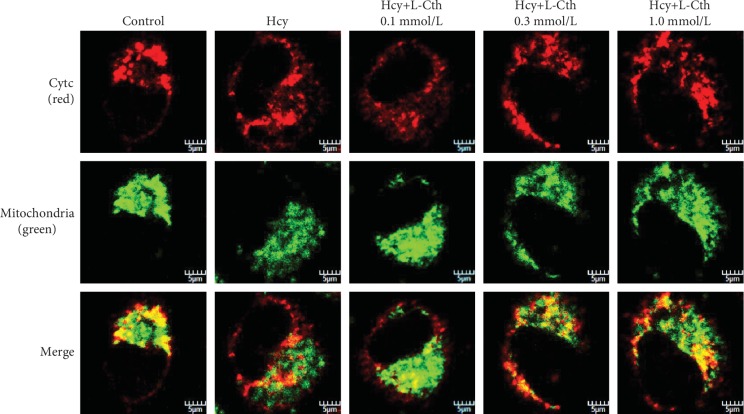
Effect of L-Cth on cytochrome c (cytc) protein expression and distribution in Hcy-treated HUVECs detected by immunofluorescence microscopy, with red fluorescence indicating cytc and green indicating mitochondria (magnification, ×600; scale bar: 5 *μ*m). L-Cth: L-cystathionine.

To investigate whether MPTP opening had an effect on cytc release, the immunofluorescence method was used to detect the leakage of cytc from the mitochondrion into the cytoplasm in HUVECs. Compared with the control group, 500 *μ*mol/L Hcy treatment significantly downregulated cytc protein expression in mitochondria of HUVECs and markedly increased cytoplasmic cytc. The pretreatment with 0.1 mmol/L L-cystathionine had no effect on the cytc expression in the mitochondrion and cytoplasm. Interestingly, 0.3 and 1.0 mmol/L L-cystathionine significantly upregulated cytc expression in the mitochondrion, respectively, but downregulated cytc expression in the cytoplasm, demonstrating the inhibiting effect on the leakage of cytc from the mitochondrion into the cytoplasm (Figure 5).

### 3.8. L-Cystathionine Inhibits Caspase-9 Activation Induced by Hcy in HUVECs

**Figure 6:**
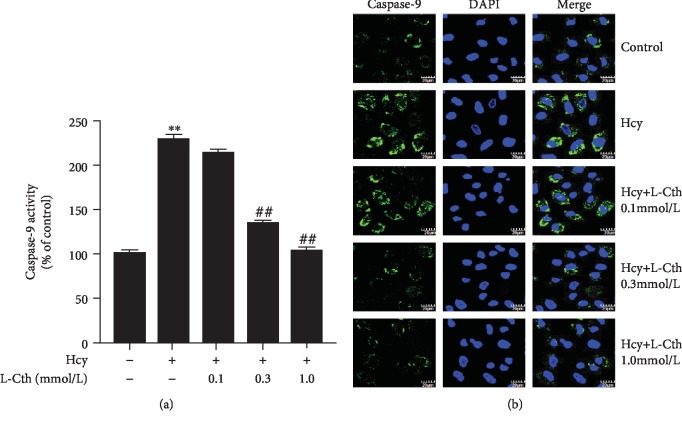
Effect of L-Cth on caspase-9 activities in Hcy-treated HUVECs. (a) Quantitative analysis of caspase-9 activities measured with a caspase-9 activity colorimetric kit. (b) Caspase-9 activity detected with living cell caspase-9 Fluo-staining kit (magnification, ×600; scale bar: 20 *μ*m). Data are presented as mean ± SEM. ^∗∗^*P* < 0.01 versus control group; ^##^*P* < 0.01 versus Hcy group. L-Cth: L-cystathionine.

The colorimetry results showed that 500 *μ*mol/L Hcy significantly increased the caspase-9 activity, whereas the pretreatment with 0.3 and 1.0 mmol/L L-cystathionine significantly inhibited the caspase-9 activity, respectively (Figure 6(a)). The fluorescence results showed that the green fluorescence intensity was significantly strengthened in the Hcy group compared with the control group. However, the fluorescence intensity was significantly weakened with the pretreatment of 0.3 and 1.0 mmol/L L-cystathionine, respectively, suggesting the inhibition of caspase-9 activities (Figure 6(b)). Both methods indicated that 0.1 mmol/L L-Cth has no effect on the caspase-9 activation (Figure 6).

### 3.9. BTSA1 Abolished the Inhibitory Effect of L-Cystathionine on Apoptosis Induced by Hcy in HUVECs

**Figure 7:**
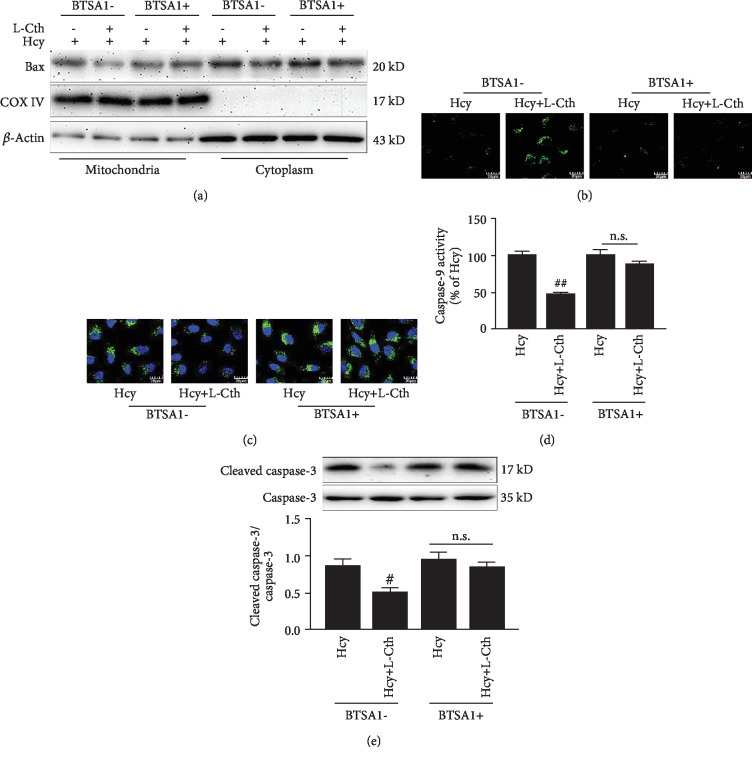
Effect of L-Cth on Bax expression, MPTP opening, caspase-9 activity, and cleaved-caspase3 expression in Hcy-treated HUVECs when administering BTSA1 in advance. (a) Bax expression analyzed by western blotting. (b) Changes of MPTP opening in HUVECs (magnification, ×600; scale bar: 20 *μ*m). (c) Caspase-9 activity detected with the living cell caspase-9 Fluo-staining kit (magnification, ×600; scale bar: 20 *μ*m). (d) Quantitative analysis of caspase-9 activities measured with the caspase-9 activity colorimetric kit. (e) Cleavage of caspase-3 analyzed by western blotting. Data are presented as mean ± SEM. ^#^*P* < 0.05 versus Hcy group; ^##^*P* < 0.01 versus Hcy group; n.s.: no significance; L-Cth: L-cystathionine.

To further demonstrate that L-cystathionine exerted antiapoptotic effects through the Bax pathway, the HUVECs were pretreated with BTSA1, a Bax agonist that promotes Bax translocation to mitochondria but has no effect on its expression [14]. Results showed that in the absence of BTSA1, 0.3 mmol/L L-cystathionine inhibited the translocation of Bax to mitochondria (Figure 7(a)), the opening of MPTP, and the activation of caspase-9 and caspase-3 (Figures 7(b)–7(e)), thereby antagonizing apoptosis induced by Hcy in HUVECs. However, after the pretreatment with 0.625 *μ*mol/L BTSA1, L-cystathionine could no longer inhibit the translocation of Bax to mitochondria (Figure 7(a)), the opening of MPTP, and the activation of caspase-9 and caspase-3 induced by Hcy (Figures 7(b)–7(e)), suggesting that the protective effect of L-cystathionine on the Hcy-induced apoptosis in HUVECs was abolished.

### 3.10. Overexpression of Bax Abolished the Inhibitory Effect of L-Cystathionine on Apoptosis Induced by Hcy in HUVECs

**Figure 8:**
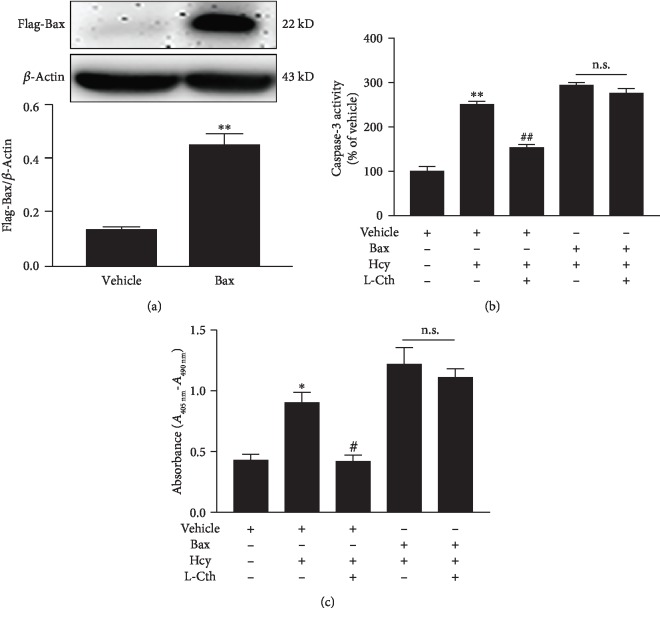
Effect of L-Cth on caspase-3 activity and apoptosis in Hcy-treated HUVECs when Bax is overexpressed. (a) Bax overexpression in HUVECs detected by western blotting. (b) Quantitative analysis of caspase-3 activities measured with the caspase-3 activity colorimetric kit. (c) Apoptosis of HUVECs measured with the Cell Death Detection ELISA^PLUS^ Kit. Data are presented as mean ± SEM. ^∗^*P* < 0.05 versus control group; ^∗∗^*P* < 0.01 versus control group; ^#^*P* < 0.05 versus Hcy group; ^##^*P* < 0.01 versus Hcy group; n.s.: no significance; L-Cth: L-cystathionine.

In order to prove if L-cystathionine plays an antiapoptotic role by inhibiting the expression of Bax, we overexpressed Bax protein with the transfection of plasmid (Figure 8(a)). Results showed that in the vehicle group, the administration of 0.3 mmol/L L-cystathionine significantly inhibited caspase-3 activation induced by Hcy, whereas in the overexpression group, L-cystathionine failed to inhibit caspase-3 activation (Figure 8(b)). The ELISA results also showed that 0.3 mmol/L L-Cth in the vehicle group could inhibit the apoptosis of endothelial cells induced by Hcy, while in the Bax overexpression group, the antiapoptotic effect of L-cystathionine was abolished (Figure 8(c)).

## 4. Discussion

Our results demonstrated for the first time that L-cystathionine could inhibit Hcy-induced mitochondria-mediated apoptosis of HUVECs via restraining the expression and translocation of Bax, increasing mitochondrial membrane potential, inhibiting MPTP opening, suppressing cytc release from mitochondria into the cytoplasm, and reducing caspase-9 activities and protein expression.

The maintenance of homeostasis in the cardiovascular system is a complex process, and VECs are critical in this process. VECs regulate vascular tone by secreting nitric oxide synthase, endothelin-1, etc., and when they are damaged, they can secrete various inflammatory mediators such as TNF-*α*, IL-1, and IL-6 [17–20]. Many studies have been conducted to explore the mechanism of VEC damage caused by Hcy. The discovery of circulating endothelial cells in the plasma of patients with severe hyperhomocysteinemia suggests that the abnormal vascular function caused by high homocysteine level is not only due to the loss of NO bioavailability but also associated with VEC apoptosis. Studies have shown that pathologically relevant levels of homocysteine can induce apoptosis of cultured endothelial cells by regulating endoplasmic reticulum stress and unfolded protein responses [21]. In order to treat hyperhomocysteinemia, in addition to supplementing group B vitamins, a large number of studies have been carried out. Wei et al. found that hydrogen sulfide attenuated Hcy-induced cardiomyocytic endoplasmic reticulum stress in rats [22]. Liu et al. found that epigallocatechin gallate (EGCG) inhibited vascular endothelial cell apoptosis by regulating the PI3K/Akt/eNOS signaling pathway [23]. Jin et al. found that paclitaxel inhibited endoplasmic reticulum stress and endothelial cell apoptosis by regulating Nrf2-dependent HO-1 expression [24]. However, up to now, there has no effective medication for vascular damage caused by hyperhomocysteinemia. L-cystathionine is a key intermediate in the sulfur transformation process [25]. So far, we know little about the biological effects of L-cystathionine. Preliminary studies suggested that L-cystathionine might have protective effects in many aspects, including significantly reducing superoxide anion produced by human leukocytes and preventing hepatic steatosis and acute tubular necrosis caused by endoplasmic reticulum stress [26, 27]. It can also reduce the apoptosis of U937 cells and HepG2 cells by inhibiting the excretion of glutathione [28]. It is noteworthy that L-cystathionine inhibits the macrophage apoptosis induced by ox-LDL [29]. Therefore, we speculated that L-cystathionine may protect against vascular damage caused by Hcy. In our studies, we first found that L-cystathionine antagonized homocysteine-induced mitochondria-dependent apoptosis of vascular endothelial cells. It is of great significance in the understanding of the interaction among the metabolites in the methionine metabolic pathway in keeping the vascular homeostasis and providing a novel approach for the prevention and therapy of apoptosis-related cardiovascular diseases.

Firstly, we evaluated the cytotoxicity of Hcy in HUVECs by measuring cell viability and LDH release. Results showed that the treatment of HUVECs with 500 and 1000 *μ*mol/L Hcy significantly suppressed the cell viability and increased the leakage of LDH. Therefore, we selected 500 *μ*mol/L Hcy as a working concentration to investigate whether L-cystathionine has protective effects on Hcy-induced HUVEC injury. We used the concentrations of L-cystathionine with reference to the previous studies [12, 26–29]. We found that 0.3 mM and 1 mM L-cystathionine significantly increased cell viability and inhibited LDH leakage and apoptosis induced by Hcy in HUVECs. Caspase-3 is an apoptosis performer in cells [30]. When cells are exposed to apoptotic stimuli, caspase-3 can be activated by the mitochondria-mediated caspase activation pathway to form cleaved caspase-3 which in turn causes apoptosis. Our experiments showed that Hcy treatment increased cleaved caspase-3 expression, while L-cystathionine reduced cleaved caspase-3 expression. All of the above results demonstrated that L-cystathionine antagonized Hcy-induced apoptosis in HUVECs.

There are many ways mediating apoptosis, and the mitochondria-mediated apoptotic pathway is the classical one. However, it is still unclear whether L-cystathionine acts through the mitochondrial apoptotic pathway. Considering that mitochondria are one of the main sites producing oxygen free radicals, abnormalities in the structure and function of mitochondria would lead to an increase in oxygen free radical generation and a decrease in removal. Previous studies have shown that Hcy increases mitochondrial oxygen free radical production, while excessive oxygen free radicals can activate mitochondria-mediated apoptosis. Therefore, we designed experiments to study the impact of L-cystathionine on the production of mitochondrial oxygen free radicals. The results indicated that L-cystathionine inhibited the production of mitochondrial oxygen free radicals caused by Hcy. Studies have shown that caspase-9 is an apoptotic promoter on the mitochondrial apoptotic pathway, which can activate caspase-3 [31]. Therefore, we detected the activation of caspase-9 by colorimetry and the fluorescence assay. The results showed that L-cystathionine inhibited the activation of caspase-9 induced by Hcy. Previous studies have shown that DNA-damaging agents activate the mitochondrial apoptotic pathway by inducing the release of cytc. Cytc, a peripheral protein of the mitochondrial inner membrane, functions as an electron shuttle. Once released into the cytosol, cytc would cause the activation of caspase-9 [32]. We used immunofluorescence to detect the leakage of cytc. The results showed that the release of cytc to the cytoplasm was increased under the exposure to Hcy, and interestingly, the leakage of cytc to the cytoplasm was significantly reduced after the administration of L-cystathionine.

The mechanism by which L-cystathionine inhibits the leakage of cytc is unclear. Stabilization of mitochondrial membrane potential is essential for maintaining mitochondrial function. A decrease in mitochondrial membrane potential implies that the cell is in the early stages of apoptosis, meanwhile cytc in the mitochondria leaks out into the cytoplasm [33]. Thus, we examined the changes in mitochondrial membrane potential and found that L-cystathionine antagonized the decrease in mitochondrial membrane potential induced by Hcy and maintained the stability of mitochondrial membrane potential. Studies showed that in association with a decrease in mitochondrial transmembrane potential, excessive reactive oxygen species- (ROS-) triggered apoptosis is mediated by MPTP [34]. MPTP is composed of a variety of protein molecules existing between the mitochondrial inner and outer membranes and is a nonspecific channel whose molecular composition has not been fully studied [35]. Under physiological conditions, MPTP is periodically opened, nonselectively allowing water and small molecules to pass through, maintaining the electrochemical balance in the mitochondria, while protons can freely pass through the mitochondrial inner membrane, causing a potential difference inside and outside the mitochondria to form a stable mitochondrial membrane potential [36]. Therefore, we examined the opening of MPTP and found that Hcy did increase the opening of MPTP, while L-cystathionine inhibited the opening of MPTP.

Studies have shown that the opening and closing of MPTP are closely related to the concentration ratio of Bcl-2 and Bax on the outer membrane of the mitochondria. Both Bcl-2 and Bax belong to the Bcl-2 family and are often expressed in tissue cells. Bcl-2 is an antiapoptotic protein. Bax is a proapoptotic protein, and some scientists even refer to the ratio of the two as an “apoptosis switch” [37]. The decreased Bcl-2/Bax ratio promotes MPTP opening, leading to apoptosis [38]. We examined the effects of L-cystathionine on Bcl-2 and Bax. Results showed that L-cystathionine inhibited the expression of Bax protein, increased the ratio of Bcl-2/Bax, and inhibited the translocation of Bax to mitochondria, thereby antagonizing apoptosis. These results demonstrated that L-cystathionine antagonized the apoptosis of vascular endothelial cells induced by Hcy by regulating the mitochondrial apoptosis pathway.

To examine if L-cystathionine inhibited Hcy-induced apoptosis of vascular endothelial cells by targeting the Bax pathway, we used Bax agonist and Bax overexpression, respectively, in the experiment. Results showed that BTSA1 or overexpression of Bax successfully prevented the inhibitory effect of L-cystathionine on the Hcy-induced opening of MPTP and the activation of caspase-3 and caspase-9. Bax agonist or Bax overexpression subsequently blocked the inhibition of L-cystathionine on Hcy-induced apoptosis in HUVECs as well. The above facts demonstrated that L-cystathionine antagonized vascular endothelial cell apoptosis by inhibiting the expression of Bax and its translocation to mitochondria.

This study still has some limitations. The molecular mechanism for L-cystathionine-mediated antiapoptosis via the mitochondrial pathway requires further study. In particular, it is necessary to explore the biologic protective role of cystathionine in endothelial injury induced by hyperhomocysteinemia in animal models. However, our present studies for the first time showed that L-cystathionine inhibited homocysteine-induced mitochondria-dependent apoptosis of vascular endothelial cells by inhibiting the expression and translocation of Bax, which would be of great value in further exploration of the potential therapeutic targets to protect vascular injury.

## Data Availability

The data used to support the findings of this study are available from the corresponding author upon request.
